# Strain-Mediated Giant Magnetoelectric Coupling in a Crystalline Multiferroic Heterostructure

**DOI:** 10.1021/acsami.0c18777

**Published:** 2021-01-27

**Authors:** Adrián Begué, Miguel Ciria

**Affiliations:** † Instituto de Nanociencia y Materiales de Aragón (INMA), CSIC-Universidad de Zaragoza, Zaragoza 50009, Spain; ‡ Departamento de Física de la Materia Condensada, Universidad de Zaragoza, Zaragoza 50009, Spain

**Keywords:** magnetoelectric coupling effect, muliferroic, heterostructure, nonvolatile, magnetoelastic coupling

## Abstract

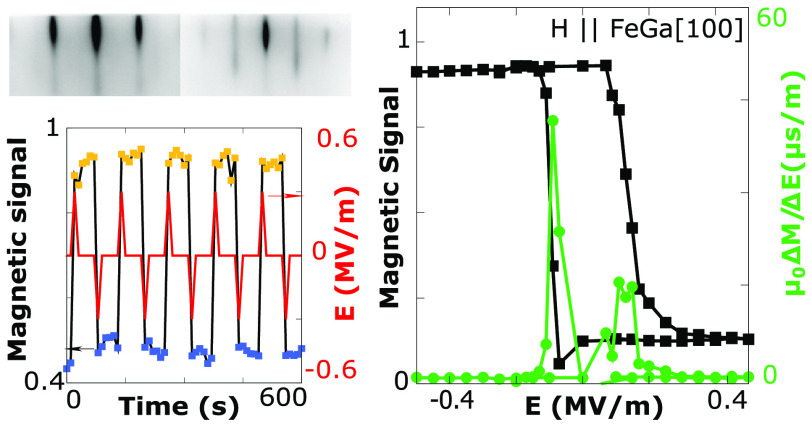

Multiferroic heterostructures based on the strain-mediated mechanism present ultralow heat dissipation and large magnetoelectric coupling coefficient, two conditions that require endless improvement for the design of fast nonvolatile random access memories with reduced power consumption. This work shows that a structure consisting of a [Pb(Mg_1/3_Nb_2/3_)O_3_]_0.7_-[PbTiO_3_]_0.3_ (001) substrate on which a crystalline FeGa(001)/MgO(001) bilayer is deposited exhibits a giant magnetoelectric coupling coefficient of order 15 × 10^–6^ s m^–1^ at room temperature. That result is a 2-fold increment over the previous highest value. The spatial orientation of the magnetization vector in the epitaxial FeGa film is switched 90° with the application of electric field. The symmetry of the magnetic anisotropy is studied by the angular dependence of the remanent magnetization, demonstrating that poling the sample generates a switchable uniaxial magnetoelastic anisotropy in the film that overcomes the native low 4-fold magnetocrystalline anisotropy energy. Magnetic force microscopy shows that the switch of the easy axis activates the displacement of domain walls and the domain structures remain stable after that point. This result highlights the interest in single-crystalline structures including materials with large magnetoelastic coupling and small magnetocrystalline anisotropy for low-energy-consuming spintronic applications.

## 1 Introduction

The processing of information requires very efficient devices with low-energy consumption, a goal that is hampered if electric current is used to switch nonvolatile states.^1,2^ This request has motivated the evolution of the vintage idea of a material with interconnected magnetic and electric capacities^3^ into sophisticates magnetoelectric (ME) heterostructures encompassing components with enhanced ferroelectric (FE) and ferromagnetic (FM) properties.^4−7^ For the latter structures, the ME coupling strength can be three or more orders of magnitude^8−10^ higher than that for single-phase materials^11,12^ and the active control of the magnetic state by the FE part of the structure can be easily achieved at room temperature.^13^ Several mechanisms are capable of controlling the magnetization *M* without magnetic field or electric current.^9,14−16^ One of them is based on the strain transferred from the FE crystal to a FM film, which generates a uniaxial magnetic anisotropy through the magnetoelastic coupling effect.^17−19^ Other mechanisms are based on phenomena located at the FE-FM interface: modification of the population of spin-up and spin-down electron density of states^20^ and voltage-driven oxygen migration and modification of the oxide ferromagnet.^21^ The strain-transfer mechanism shows lower heat dissipation per switching cycle^16^ and presents larger magnetoelectric coupling parameter α_E_ than any other coupling mechanism.^9^

Materials with large electrostriction, such as the relaxor FE compound [Pb(Mg_1/3_Nb_2/3_)O_3_]_(1–*x*)_-[PbTiO_3_]_*x*_ (*x* ∼ 0.3) (PMN–PT),^22^ are used to induce strain in magnetic thin films with significant magnetoelastic coupling. Many of these films are amorphous^17,23^ or polycrystalline^24−29^ to diminish the native large magnetocrystalline anisotropy that can conceal the effect due to the FE domain switching, usually detected by 90° easy axis switching.^30^ Studies on crystalline films present also strain induced effects^8,31−34^ and dependencies of the cubic magneto-crystalline constants on the electric field *E*.^35,36^ However, the larger values of α_E_ for strain-mediated coupling are below 1 × 10^–5^ s m^–1^.^9,10,29^

This work reports on a hybrid ME structure including a crystalline layer of the magnetostrictive FeGa alloy that exhibits values for α_E_ in the range of 1 × 10^–5^ s m^–1^ at room temperature. This result is achieved by the activation of a uniaxial magnetoelastic anisotropy on top of the native small cubic magnetocrystalline anisotropy. The crystalline magnetoelectric structure is obtained by the deposition of a thin layer of MgO (001) on a PMN–PT(001) substrate as a buffer layer. The Fe_100–*z*_Ga_*z*_ alloy, with *z* around 20, has been proposed as magnetic component in ME devices to achieve the switching between defined magnetic states.^37^ The reason is the giant tetragonal magnetostriction,^38,39^ which can reach a value more than 50 times that of pure iron with appropriate doping.^40^ The cubic magnetocrystalline energy in Fe_80_Ga_20_ is small with values for the first-order anisotropy coefficient *K*_1_ around −10 kJ m^–3^,^41,42^ clearly below that the value for pure iron of 48 kJ m^–3^. The change in the sign of *K*_1_ means that for (001) thin films the easy axis moves from the ⟨100⟩ to the ⟨110⟩ in-plane directions as the gallium content increases. Thus, FeGa crystalline films with intrinsic small cubic magnetic anisotropy and large magnetostriction can improve the performances of magnetoelectronic devices based on converse magnetoelectric mechanisms.

## 2 Results and Discussion

The multiferroic heterostructure consists of a PMN–PT(001) FE substrate, coated with a crystalline MgO(001) seed layer (≈ 3 nm in thickness) for a FeGa(001) magnetic layer approximately 15 nm thick and a Mo overcoat 2 nm thick. The Mo/FeGa/MgO trilayer was grown by molecular beam epitaxy. The crystal orientation of these layers is observed in situ by reflection high energy electron diffraction (RHEED), and ex situ by aberration-corrected scanning transmission electron microscopy. The resulting epitaxial relationships are FeGa[110] ∥ MgO[100] ∥ PMN–PT[100], and FeGa[100] ∥ MgO[110] ∥ PMN–PT[110] (for details of the film growing and its characterization, see the Supporting Information).

**Figure 1:**
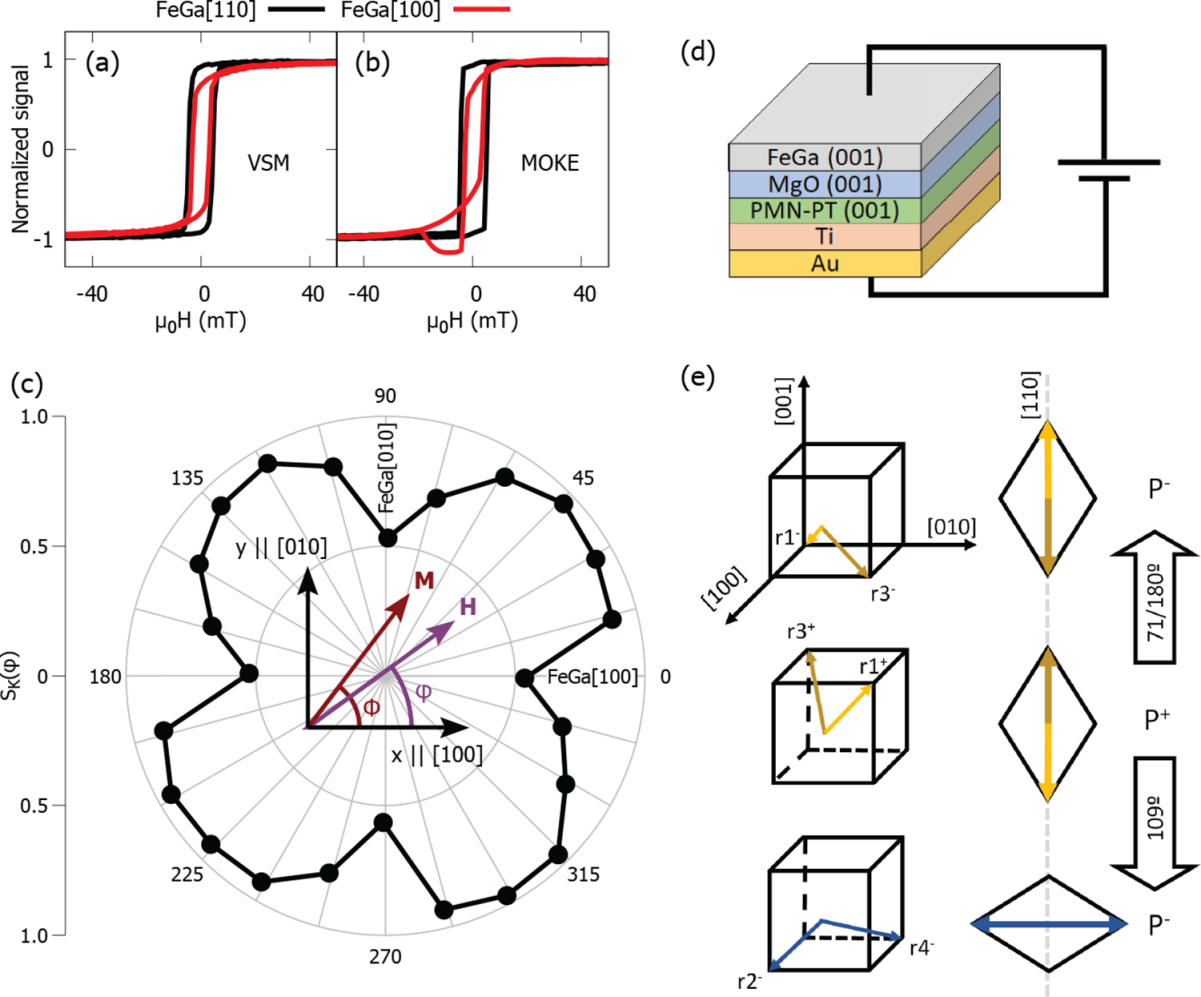
Hysteresis loops for the as-grown FeGa(001) film performed by means of (a) VSM magnetometry and (b) Kerr magnetometry. The magnetic field is applied along the [110] and [100] in-plane directions. The magnetic signals were normalized by the value obtained at saturation at 100 mT. (c) Polar plot of the angular dependence of the Kerr signal obtained at remanence normalized by the saturation value *S*_K_(φ). The coordinate system and the angle definition used in the text is shown as inset. (d) Schematic drawing of the device fabricated to apply electric field. (e) Sketch of the ferroelectric switching mechanisms with *E* applied along the [001] direction.

Figure 1a shows *MH* loops of the as-grown film carried out by vibrating sample magnetometry (VSM) with *H* applied along the [100] and [110] FeGa directions. These loops suggest the presence of a 4-fold magnetic anisotropy (see Supporting Information). It is observed that the magnetic behavior of FE-FM systems can be inhomogeneous because of the presence of several FE switching modes.^43,44^ Thus, the measurement of the magnetic response of the whole film averages the variation of *M* under the application of *E* causing a decrement in α_E_. To avoid this effect, we measured the magnetic behavior of the film employing a MOKE magnetometer with a laser focus spot diameter in the micrometer range. Figure 1b displays the Kerr rotation signal for loops taken on the as-grown film, which serve as reference for the effect of poling the FE crystal. The loop with *H* along the [001] direction shows features that can be explained by the presence of quadratic contributions to the Kerr rotation (see the Supporting Information). The azimuthal dependence of the remanent magnetization obtained from the MOKE measurements normalized by the signal at the saturation field, *S*_K_(φ), is presented in Figure 1c as a function of the φ angle between *H* and the FeGa [100] direction. The symmetry of this curve can be associated with a dominant 4-fold anisotropy term in the film plane. Also, assigning the easy (hard) directions with high (low) *S*_K_ determines that the [110] direction is the easy axis (EA) and the [100] one the hard axis (HA), a fact also observed in FeGa films with similar composition grown directly on MgO(001) crystals.^42^

**Figure 2:**
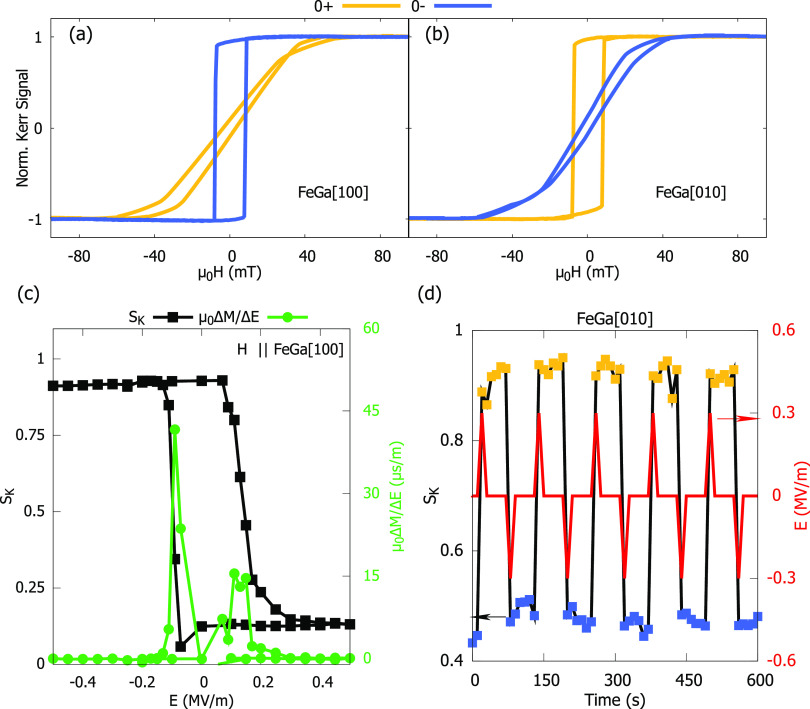
Hysteresis loops for the FeGa(001) film obtained by means of MOKE magnetometry in the film at 0 V m^–1^ after applying positive (0+) and negative (0−) electric field. The magnetic field is applied along (a) [100] and (b) [010] FeGa directions. (c) Variation of *S*_K_ and the magnetoelectric coupling coefficient with the electric field. (d) Normalized Kerr rotation signal vs time curve showing five cycles of repeatable high and low magnetization states switched by pulses of electric field.

To apply electric field along the [001] direction of the PMN–PT(001) crystal, we deposited a Ti/Au bilayer on the uncovered PMN–PT surface (Figure 1d). An initial poling of the crystal was performed by application of *E* = 0.6 MV m^–1^. The symbols 0+ and 0– are used to define the state of remanent polarization after applying electric field with positive and negative polarity, respectively, and |*E*| = 0.3 MV m^–1^, which is large enough to switch *P* (Figure 1e). Kerr loops were performed at several areas of the film to identify the homogeneity of the magnetic behavior in the sample.^43^ The most compelling result is shown in Figure 2a, b, where Kerr rotation loops with *H* along the FeGa [100] and [010] directions for 0+ and 0– states are compared. These loops demonstrate that the EA and HA switched 90° by the action of *E*. Notice also that *S*_K_ for the HA loop abates when compared with the value obtained from the HA loop taken before poling (Figure 1b). These results indicate that the uniaxial magnetic anisotropy emerged with the application of *E* can be controlled externally. However, for other regions of the film, the configuration of the easy and hard direction remains fixed under the application of electric field with positive or negative bias. The inhomogeneous magnetic response of the FeGa film has been also observed in amorphous CoFeB thin films^43^ and mesoscopic disc.^44^ In any case, new magnetic anisotropies are requested to explain the observed behavior.

The *S*_K_ vs *E* dependency shown in Figure 2c was obtained from Kerr rotation loops (see the Supporting Information) performed with *H* applied along the FeGa [100] direction at fixed values of *E*. The large jumps of *S*_K_(*E*) observed for *E* ∼ 0.15 MV m^–1^ and *E* ∼ −0.1 MV m^–1^ present an asymmetry with respect to *E*. An explication for the shift in the *S*_K_(*E*) curve could be ascribed to the inhomogeneous FE domain switching through the structure. The ferromagnetic exchange coupling interaction across the boundary of areas with switchable and fixed EA favors parallel orientation of M. The effect of this interaction is an unidirectional anisotropy that can enforce or arrest the inversion of *M* in the reversible area and is observed as a shift of the *S*_K_(*E*) loop.

The converse magnetoelectric coupling coefficient α_E_, calculated from the *S*_K_(*E*) data as μ_0_Δ*M*/Δ*E* (μ_0_ is the vacuum permeability), is also shown in Figure 2c. The value of α_E_ around *E* = 0.15 MV m^–1^ is 15 × 10^–6^ s m^–1^ and is, to the best of our knowledge, the largest α_E_ observed for any kind of coupling mechanism:^9,10^ α_E_ of order 8 × 10^–6^ s m^–1^ is obtained for optimized structures of amorphous films grown on relaxor substrates,^10^ whereas for oxide magnetic layers, α_E_ can be on the order of 1 × 10^–7^ s m^–1^.^8^ For the interfacial oxidation coupling mechanism, α_E_ decreases to values in the range of 1 × 10^–8^ s m^–1^.^9^ We note that α_E_ ∼ 40 × 10^–6^ s m^–1^ at *E* = −0.1 MV m^–1^, although the switching rate can be enhanced by the orientation of *M* in neighbor domains. The Kerr loops used to calculate α_E_ were obtained in an area with EA switching. In other regions, the maxima of α_E_ could peak at different *E* or have smaller magnitude because the Kerr signal integrates the response from domains that switch at different values of *E* (see the calculation of α_E_ for a polycrystalline film in the Supporting Information).

A large change in the value of *S*_*K*_(φ) induced by the aplication of electric field can be useful for applications. An important issue is the observation of reproducible changes in the magnetic response through the application of pulses of *E* with alternating polarity. Figure 2d has been obtained at *H* = 0 mT after saturating the film along a direction close to one FeGa [010] axis in the area that shows EA switching; it presents repeatable jumps between two clearly different values, for a set of 10 pulses with |*E*| = 0.3 MV m^–1^. The Kerr rotation signal changes markedly and abruptly when the electric field pulse oscillates between positive and negative values back and forth. It is relevant that the magnitude of the switching between two states is large and around a fixed value. One-hundred eighty degree switching, from + *M* to −*M*,^45^ can also be obtained with the assistance of external magnetic field of 3 mT (see the Supporting Information).

**Figure 3:**
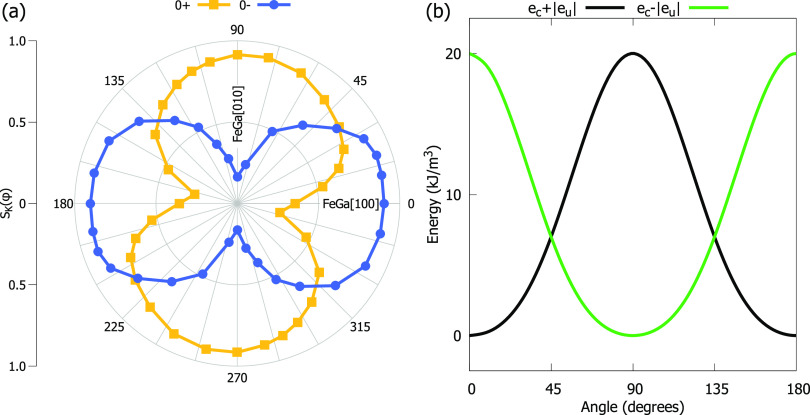
(a) Polar plot of *S*_K_(φ) for the 0– and 0+ states obtained by rotation the H with a quadrupole magnet. (b) Sum of the uniaxial *e*_u_ = *K*_me_sin^2^ϕ and cubic anisotropy energies *e*_c_ = *K*_1_  sin^2^ϕcos^2^ϕ as a function of the azimuth angle for |*K*_me_/*K*_1_| = 1.67, the minimum and maximum shift 90° by changing the sign of *K*_me_.

*S*_K_(φ) illustrates the modification of the magnetic energy landscape occurring with the application of electric field. To avoid the incertitude in the Kerr signal due to the location of the laser spot during the rotation on an inhomogeneous FE substrate, the angular position of the magnetic field is modified by the use of a quadrupole magnet^46^ (see the Supporting Information). The resulting polar plots of *S*_K_(φ) for the 0+ and 0– states are shown in Figure 3a. These curves display a clear two-fold symmetry, obvious differences with respect to the 4-fold *S*_K_(φ) data obtained before poling the substrate and demonstrate the switching of 90° between the easy and hard directions by the application of *E*.

**Figure 4:**
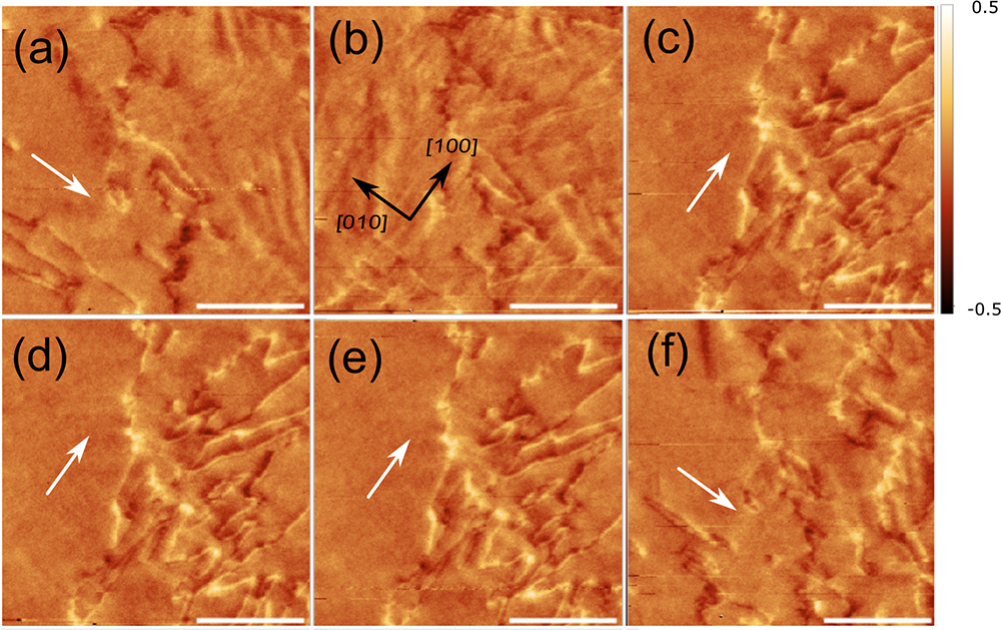
Magnetic force microscopy images taken on the film at different values of electric field on the same area: (a) 0+, (b) −0.08 MV/m, (c) −0.14 MV/m, (d) −0.2 MV/m, (e) 0–, and (f) 0+. The arrows in panel b stand for the FeGa [100]/[010] in-plane directions. The white arrows indicate the magnetic easy axis. The units of color code bar are degrees. Scale bar length 10 μm.

Active control of magnetic domain walls (DWs)^47−50^ is a matter of interest for practical applications.^51^ Magnetic force microscopy is used to directly observe the displacement and stability of the magnetic domain structure under the application of electric field without the presence of magnetic field except for that emanating from the tip. The starting magnetic domain structure was obtained from a 0+ state with the application of magnetic field along the hard direction. Figure 4a shows the magnetic image with contrast due to the presence of DWs because *M* lays in the film plane. Figure 4b was taken at *E* = −0.08 MV m^–1^, which is around the value for which a large jump of *S*_K_(*E*) is observed (Figure 2c). Compared with image of Figure 4a, clear changes are present at *E* = −0.08 MV m^–1^: sets of lines appear in areas without features while some DWs do not move but others disappear. The increment of the magnetic contrast of the film, with lines and other features on the whole area, could indicate a nonhomogeneous switching of *P*, which causes misalignment of *M*, also in a nonhomogeneous fashion, by the stresses arisen from the FE domains. Increasing the strength of *E* to −0.14 MV m^–1^, see Figure 4c, changes the landscape of the domain structure to a configuration that remains stable for *E* = −0.2 MV m^–1^ (Figure 4d) as well as for the 0– state (Figure 4e). For this set of three images, only minor changes are observed in the geometry and position of the DWs probably induced by the magnetic tip. A second application of *E* with positive bias (0+ state) modifies the domain configuration as is shown in Figure 4f. However, the domain configurations of the 0+ states (Figure 4a, f) share the presence of domain walls that are quite parallel to the easy axis, such as those in the bottom left corner near the white arrow.

### 2.1 Analysis

The angular dependencies of *S*_K_(φ) presented in Figures 1c and 3a suggest the activation of a uniaxial anisotropy contribution that overcomes the cubic anisotropy term. The strain-induced magnetoelastic contribution is the only term considered here, and the magnetic energy density, with the assumption that *M* lies on the film plane, can be written as *e*(ϕ) = *K*_1_sin^2^ϕcos^2^ϕ – *B*_1_(ϵ_*xx*_ – ϵ_*yy*_) sin^2^ϕ + *B*_2_ϵ_*xy*_sin 2ϕ (see the Supporting Information). The Cartesian reference system is aligned with the ⟨100⟩ directions of the FeGa layer with *z* parallel to the film normal and ϕ the angle between *M* and the [100] direction. *B*_1_ and *B*_2_ are cubic magnetoelastic stress coefficients and ϵ_*xx*_, ϵ_*yy*_, and ϵ_*xy*_ in-plane strain components, which are induced by the PMN–PT substrate.

The switching of *P* between different ⟨111⟩ directions by the application of *E* on the PMN–PT(001) crystal along the [001] direction can be achieved by several modes (Figure 1e) that cause an asymmetric butterfly-like strain versus *E* curve.^52^ The ferroelectric switching of *P* by the 109° mechanism provides ferroelastic jumps that persists at remanence with a value of order 0.04%^52^ for the strain along ⟨110⟩ directions. However, the mixture of the switching modes (109° with 71° and 180° mechanisms) in the whole substrate can translate the strain to the film with values that fluctuate^17,52,53^ and modify locally the magnetic anisotropy coefficients.^54^ The proportion of polarization variants with 109° switching varies in crystals with the same nominal composition.^52^ Thus, the observation of areas with induced uniaxial anisotropy insensitive to the bias of the electric field is compatible with FE domains undergoing 71° and 180° switching modes (see Figure 1e).

The combination of Kerr and scanning electron microscopy with polarization analysis (SEMPA) measurements has linked the shape of *S*_K_(φ, *E*) to the presence of single or multidomain ferroelectric switching.^44^ In mesoscopic amorphous CoFeB discs, the rotation of 90° of both *M*, observed directly by SEMPA, and *S*_K_(φ), measured by Kerr effect, are ascribed to the 109° domain switching of a single variant. *S*_K_(φ) for the FeGa film rotates by 90° between the 0– and 0+ states, see Figure 3a., in agreement with the data reported for CoFeB amorphous discs.^44^ This result suggests that in the zone probed by the Kerr magnetometer a single FE variant undergoes a 109° switching. Thus, α_E_ can be ascribed to the activation of a uniaxial magnetic anisotropy originated in that single domain area.

Because the FeGa[100] directions are parallel to the PMN–PT[110] crystal axes, the in-plane shear distortion of the FE domains is transmitted to the FeGa(001) plane as a rectangular distortion of the square symmetry of the FeGa film along the [100] and [010] directions, with strain components ϵ_*xx*_ = −ϵ_*yy*_ and ϵ_*xy*_ = 0. Thus, the uniaxial strain-induced magnetic anisotropy is *K*_me_ sin^2^ϕ, with *K*_me_ = 2*B*_1_ϵ_*xx*_. Getting ϵ_*xx*_ = ± 0.04% and *B*_1_ ≈ −15 MPa^38,39^ (for Fe_80_Ga_20_) |*K*_me_| = 12 kJ m^–3^ with the sign coefficient oscillating from positive to negative as the rectangular distortion ϵ_*xx*_ – ϵ_*yy*_ does.

The value of *K*_1_ is estimated from the MH loops as the energy required to saturate the film along the [100] and [110] directions. Thus, taking μ_0_*M*_s_ = 1.65 T for Fe_80_Ga_20_,^39^ the value *K*_1_ ≈ −12 kJ m^–3^ is obtained. The HA loop in Figure 2a is used to obtain for the uniaxial anisotropy constant of order 20 kJ m^–3^ (using for the anisotropy field μ_0_*H*_a_ = 2*M*_s_*K*_u_ ≈ 30 mT), which is not far form the value calculated above. Managing *e*(ϕ) with ϵ_*xy*_ = 0, it can be easily shown that the minima of *e*(ϕ) depend on the ratio *K*_me_/*K*_1_. For |*K*_me_/*K*_1_| > 1 the maxima and minima are at ϕ = 0 + *n*π (*n* is an integer). Therefore, using |*K*_me_| = 20 kJ m^–3^, and *K*_1_= −12 kJ m^–3^, the *e*(ϕ) curves obtained for ± *K*_me_ are shown in Figure 3b observing that the maxima/minima are exchanged between [100] and [010] directions as a function of the sign of *K*_me_, in agreement with the experimental results.

## 3 Conclusions

A crystalline multiferroic structure with giant value for α_E_ of order 15 × 10^–6^ s m^–1^ is obtained in a full crystalline heterostructure at room temperature. The heterostructure consists of a PMN–PT(001) FE substrate, coated with a crystalline MgO(001) seed layer (≈ 3 nm in thickness) for a FeGa magnetic layer approximately 15 nm thick and a Mo overcoat 2 nm thick. The alignment of the [100]FeGa with [110]PMN–PT determines that the uniaxial easy/hard directions switch between [100] and [010] FeGa directions with the application of electric field along the [001] direction. The rectangular distortion of the square symmetry of the FeGa film acts as driving factor to activate the magnetoelastic uniaxial energy. This contribution overcomes the cubic anisotropy observed in the as-grown structure, before the initial poling of the PMN–PT crystal.

The experimental procedure used to obtain the multiferroic heterostructure presented here was not optimized to maximize the value of α_E_ but was used to demonstrate the effect of incorporating a crystalline magnetostrictive layer. Thus, it can be foreseen that systematic studies varying preparation conditions can largely improve the strength of α_E_ in multiferroic heterostructures with magnetic crystalline layers, a fundamental issue to design devices for low-energy magnetic memory technologies.

## Supporting Information Available

Methods; heterostructure growth and structural characterization (RHEED, STEM, and AFM measurements); additional data and discussions about MOKE and VSM experiments; α_E_ for a polycrystalline film; demonstration of 180° switching of *M* with the assistance of magnetic field and the derivation of the magnetic anisotropy energy (PDF)
