# Supplementary material for: Strain-Mediated Giant Magnetoelectric Coupling in a Crystalline Multiferroic Heterostructure
Source: ACS Appl Mater Interfaces. 2021 Jan 27;13(5):6778–84. doi: 10.1021/acsami.0c18777 (PMC8483440; doi:10.1021/acsami.0c18777)
Supplement: Supplementary file 1 — am0c18777_si_001.pdf [file am0c18777_si_001.pdf]

Supporting Information

Strain-Mediated Giant Magnetoelectric Coupling in a Crystalline Multiferroic Heterostructure

Adrián Begué[†,‡] and Miguel Ciria[*,†,‡]

†*Instituto de Nanociencia y Materiales de Aragón (INMA), CSIC-Universidad de Zaragoza, Spain.*

‡*Departamento de Física de la Materia Condensada, Universidad de Zaragoza, Zaragoza, Spain.*

E-mail: miguel.ciria@csic.es

1 Methods

Thin film evaporation. The structure was prepared in a DCA M600 metal deposition system equipped with a Telemark multi-pocket e-beam evaporator, a Createc high temperature effusion cell, a 15 keV RHEED system (Staib Instruments) and a quartz crystal microbalance. Base pressure in the preparation chamber was 1×10^{-10} Torr. Ga chunks (99.999 % purity), Fe pieces (99.99 % purity), Mo pellets (99.98 % purity) and MgO tablets (99.9 %) were used as source materials. The PMN-PT (001) substrate was purchased from Crystal-GmbH, it has one side epi-polished and dimensions of $12.7 \times 6.35 \times 0.5$ mm^3.

Magnetic Force Microscopy. A NT-MDT Aura platform was used to perform scanning probe microscopy measurements. Images were taken in air with commercial magnetic tips (MESP-RC-V2; Bruker Probes) with force constant k $\sim$ 5 N/m and resonance frequency $\sim$ 150 kHz. The tips are covered with a layer of cobalt-chromium alloy with nominal coercivity of 400 Oe and a magnetic moment of 10^{-13} emu. A two-pass tapping/lift mode is used to obtain the topography and the magnetic signal. MFM images in Figure 4 were obtained from the phase shift observed during the scan performed at a lift height of $\sim$ 50 nm. Also, the cantilever amplitude was one-half of that used during tapping.

Magnetometry. The measurements obtained by VSM were performed with a a commercial LakeShore 7400 series unit. MOKE measurements were obtained with a NanoMOKE®3 scanning laser MOKE magnetometer equipped with a quadrupole magnet. The longitudinal Kerr configuration uses a 50 mm focal length lens. The angle of incidence of the p-polarized laser light forms 45 degrees with the film normal. The lower value for the spot laser is 2 μm for a well focused sample. Each MOKE loop presented in this work is the average of at least 12 field cycles.

Transmission Electron Microscopy. The crystal and composition study performed by transmission electron microscopy was carried out with a FEI Titan Low-base Transmission Electron Microscope with spherical aberration corrector at the condenser lens. The cross-sectional specimen was prepared along the [100] direction of the PMN-PT(001) substrate in

a FEI Helios NanoLab 600 DualBeam focused ion beam instrument using Ga ions.

The MFM and TEM images were managed with Gwyddion[1] a free data visualization and analysis software.

2 Heterostructure growth and structural characterization

The Mo/$Fe_{80}Ga_{20}$(001)/MgO(001) structure was grown by molecular beam epitaxy (MBE). Reflection high energy electron diffraction (RHEED) is used to monitor the growth process in situ. Figure S1a-f shows images taken on the PMN-PT(001) crystal, the MgO buffer layer, and the FeGa film. Prior to the growth of the film, the PMN-PT(001) crystal was kept at 800 ^{o}C to obtain a clean surface. The patterns displayed in Figure S1a,b are obtained by rotating the sample by 45° and correspond to [100] and [110] directions, respectively. Both of them have sharp spots and Kikuchi lines indicating a clean and flat surface.

The MgO buffer film was deposited at 600 ^{o}C by using an e-beam gun and annealed at 800 ^{o}C for 2 hours. RHEED images shown in Figure S1c,d were taken on the MgO layer along the PMN-PT[100] and PMN-PT[110] directions. They repeat every 90° suggesting a plane with a 4-fold symmetry. Also, the ratio $d1/d2$ (with $d1$ and $d2$ the distances between RHEED streaks defined in Figure S1c,d) is about 1.4, the predicted value for a square lattice.

Obtaining the FeGa alloy is achieved by deposition of Fe and Ga atoms by co-evaporation using an e-beam gun and a high temperature cell at 1120 ^{o}C, respectively, with a growth rate around 0.7 nm/min and the substrate at 150 ^{o}C.[2] The RHEED images taken on the film (Figure S1e,f) indicate a crystalline structure with the distances between the RHEED streaks close to those obtained for the MgO surface. Therefore, the FeGa layers undergo a rotation of 45° of the [100] in-plane directions and the epitaxial relationships are: FeGa[110] $\|$ MgO[100] $\|$ PMN-PT[100], and FeGa[100] $\|$ MgO[110] $\|$ PMN-PT[110]. The structure was covered with a Mo layer grown by e-beam evaporation. The presence of the MgO(001) layer

is necessary to obtain crystalline structures since FeGa films deposited directory on PMN-PT(001), also at the substrate temperate of 150°C, show concentric rings in the RHEED pattern (Figure S1g) indicating a polycrystalline structure.

The misfit η between FeGa and PMN-PT and MgO is calculated considering the alignment between FeGa[100] and PMN-PT[110] or MgO[110]. The lattice parameters are: for the FeGa alloy $a_{FeGa} \sim 0.290$ nm,[2] for PMN-PT $a_{PMN-PT} \sim 0.402$ nm,[3] and for MgO $a_{MgO} \sim 0.421$ nm. For the interface FeGa/MgO, $\eta_{FeGa/MgO} \left[= \left(\sqrt{2}a_{FeGa} - a_{MgO}\right)/a_{MgO}\right]$ is $\sim$ -2.6% and for FeGa/PMN-PT, $\eta_{FeGa/PMN-PT} \sim 2\%$. Thus, although the absolute value of the misfits are similar and around 2.3%, a crystalline layer of FeGa is only obtained on the MgO(001) surface.

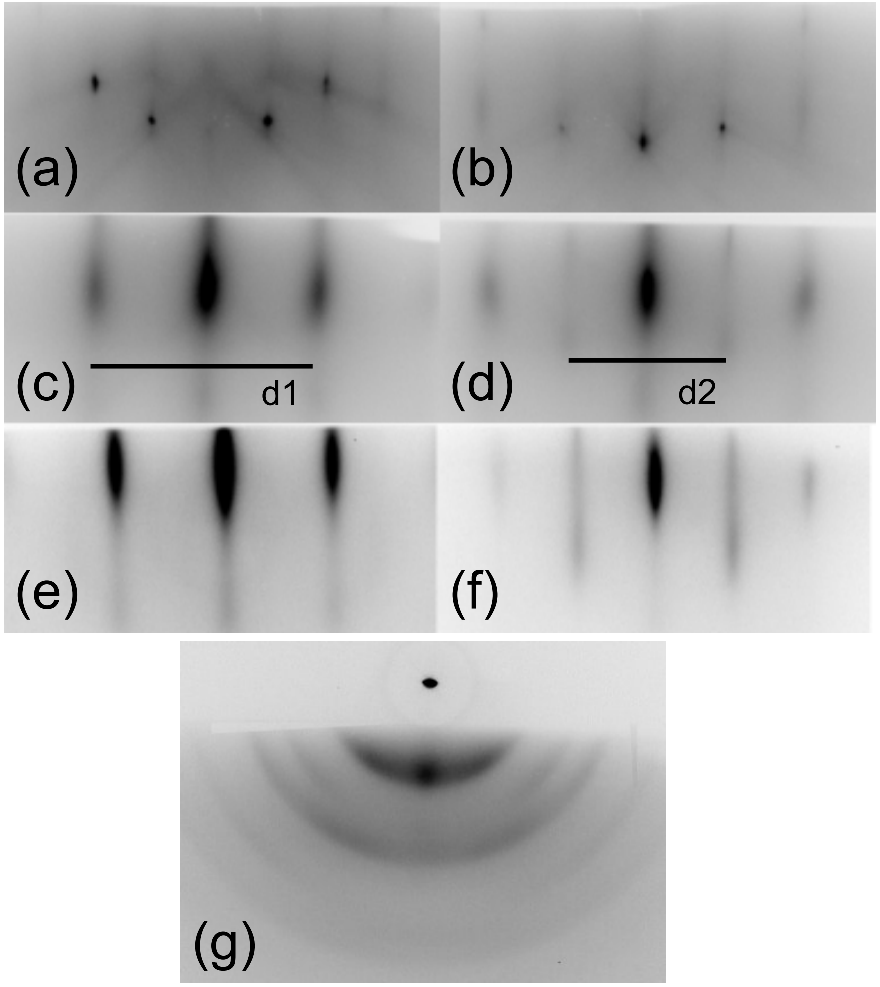

Figure S1: RHEED patterns in three steps of the preparation of the magnetoelectric epitaxial structure. (a-b) Clean PMN-PT(001) substrate, (c-d) MgO layer (e-f) FeGa film. Sets of images for the left (a, c and e) and right (b, d and f) columns were taken, respectively, along PMN-PT[100] and PMN-PT[110] azimuthal directions. (g) RHEED pattern for a FeGa film on a PMN-PT (001) crystal at 150° C.

The sample was characterized ex situ by aberration-corrected scanning transmission electron microscopy in combination with electron energy-loss spectroscopy (STEM-EELS) and atomic force microscopy (AFM). Figure S2a shows a representative high-angle annular dark-field (HAADF) image of the structure along the [100] zone axis of the PMN-PT substrate.

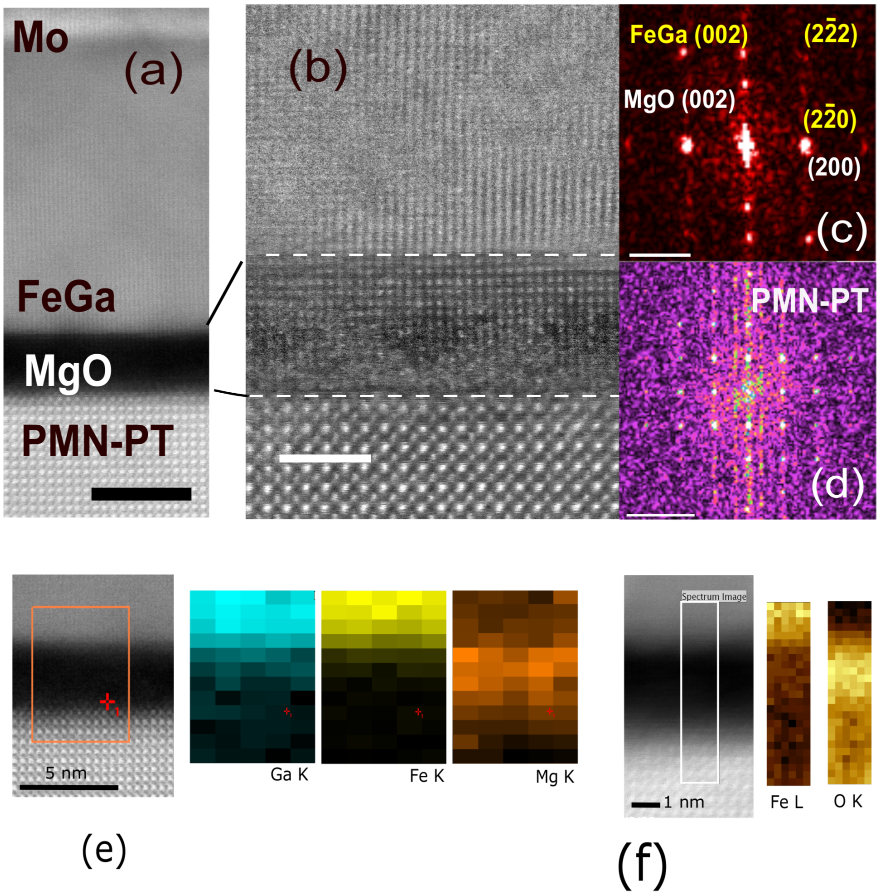

Figure S2: (a) Representative high-resolution spherical aberration-corrected STEM image of the structure viewed along the [100] zone axis direction of the PMN-PT substrate. Scale bar length 5 nm. (b) Enlarged area of the FeGa/MgO/PMN-PT structure. Scale bar length 2 nm. Fast Fourier Transforms of (c) FeGa + MgO and (d) PMN-PT areas. Scale bar length 5 nm^{-1}. (e) EDS and (f) EELS maps obtained on the areas marked with a rectangles on the HTEM image. The K-edge signal for Ga (blue) and Fe (yellow) and Mg (orange) was used to determine the EDS maps. Fe L-edge and O K-edge was selected to obtain the corresponding EELS maps for which brighter colour indicates higher presence of the selected element.

The intensity of the signal scales with the atomic number of the element and the substrate presents the brighter dots due to Pb columns. Above, the MgO layer appears as black stripe due to the presence of light atoms, the next layer corresponds to the FeGa block, with the Mo capping layer at the top position. Figure S2b shows a HAADF image on the area around the MgO layer taken at higher resolution with the contrast enhanced locally. The image displays atomic ordering for the MgO and FeGa layers, while for the PMN-PT crystal bright points located at the midpoints of the Pb square lattice become visible. The Fast Fourier Transform (FFT) from an area that includes FeGa and MgO is presented in Figure S2c. The MgO layer can be indexed to the [100] zone axis, while for the FeGa film the distances between spots along orthogonal directions reveal the [110] zone axis with the [1-10] direction in the film plane. The FFT for a region of the PMN-PT substrate shown in Figure S2d corresponds to the [100] zone axis. These results support the proposed relations between in-plane directions observed by RHEED. The composition maps of the structure show a sharp transition between the FeGa and MgO layers Figure S2e,f. A loss of resolution is noted at the interface between MgO and PMN-PT, which is likely due to a projection effect of the steps at PMN-PT surface that can be appreciated in the AFM image (Figure S3).

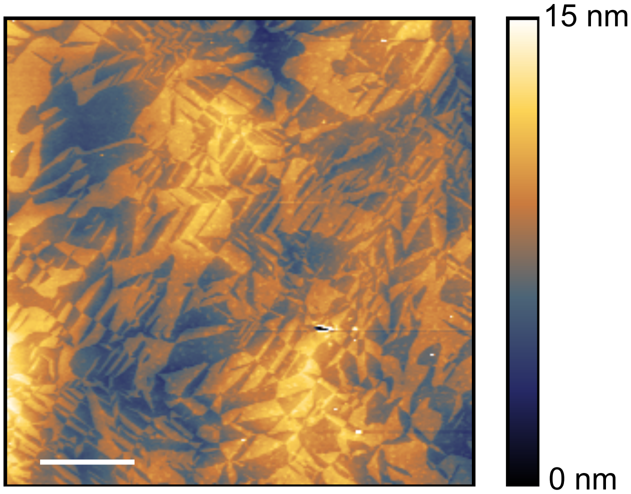

Figure S3: Atomic force microscopy image of the Mo/FeGa/MgO/PMN-PT structure, scale bar length 5 μm.

3 Magnetic characterization

3.1 VSM and MOKE loops

From the VSM measurements, see Figure 1a in the main text, the ratio M_r/M_s (M_r remanent magnetization, M_s saturation magnetization) for the MH loop with H along the FeGa[100] hard axis (HA) is ≈ 0.7. This value is in agreement with the presence of a square symmetry in the film which yields $M_r \approx M_s \cos 45^o$ if $\mathbf{M}$ moves to easy directions when H is applied along a hard $\langle 100 \rangle$ direction. The Kerr rotation MOKE loop with H along the HA, see Figure 1b, shows features described for Fe films grown on MgO: (i) one or two jumps of the Kerr rotation signal θ_K that are explained in terms of the superposition of uniaxial and cubic anisotropies[4,5] and (ii) antisymmetry parts in the hysteresis loops that indicate the presence of longitudinal and quadratic MOKE effects.[6]

3.2 Determining $\alpha_K(E)$

As indicated in the main text, $\theta_K(H)$ loops were taken at fixed values of E to determine $\alpha_K(E)$. Figure S4 shows some of these loops taken at relevant values of E after the following corrections were carried out. The raw Kerr loops show a lineal dependence with H that may be due to the presence of the Mo capping layer. A slope compensation was performed in two steps. (a) Fitting data above a cut-off field with a linear regression $a+bH$. In order to maximize the H-field interval for the fitting, a loop performed with H along the easy direction was used. (b) Subtraction of the linear contribution bH, with b obtained in step (a) for the all set of measurements. To obtain M_r, the data at zero field for the rising and decreasing magnetic field sections of the MH loop were averaged.

In order to calculate α_E, the value for the Kerr signal at saturation is assigned to $\mu_0 M_s$ = 1.65 T, the magnetization saturation value for the $Fe_{80}Ga_{20}$ alloy.[7]

$\theta_K(H)$ loops may present longitudinal and quadratic magneto-optic Kerr effects, see Figure 2b. One procedure used to extract the longitudinal Kerr contribution, proportional

to the longitudinal magnetization component, consist of adding the increasing and decreasing branches of the cycle.[8] The resulting symmetrized loops are shown in Figure S4.

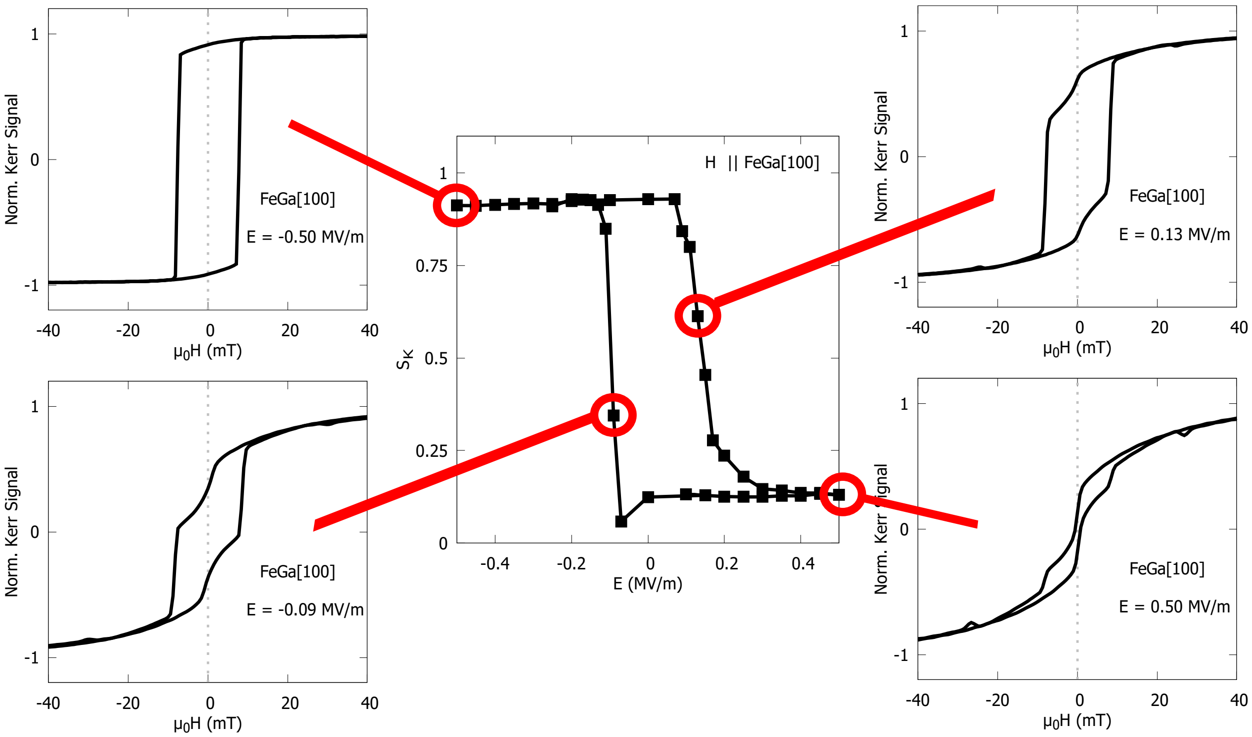

Figure S4: Magnetoelectric switch of **M** by the application of electric field.

3.3 $\alpha_K(E)$ for a polycrystalline film

$\alpha_K(E)$ is presented for a FeGa polycrystalline film (see Figure S1g) grown on a PMN-PT(001) crystal as described above. Thus, $\theta_K(H)$ loops, at fixed values of E, were taken with the magnetic field applied along a $\langle 110 \rangle$ PMN-PT in-plane direction at four different areas of the sample. Figure S5 displays $S_K(E)$ and $\alpha_K(E)$ for each zone and the result of averaging over the four regions.

This film shows nonvolatile effects, and $S_K(E)$ is doubled-valued for the four regions studied. The overall jump of the magnetization in the polycrystalline film (around 10%) is much smaller than that observed in the crystalline film (larger than 60%, see Figure S4). That is an important difference for practical applications. The sign of ΔM and α_E can be

either positive or negative for the same ΔE. $\alpha_E(E)$ presents an asymmetric behavior for the increasing and decreasing branches of the E-cycle, a result also observed in the crystalline film. The representative respond of the sample will require the average of several areas because the mesoscopic response changes from point to point. Thus, the jumps of $S_K(E)$ get smoother and the peaks of $\alpha_K(E)$, observed at particular regions, fade away.

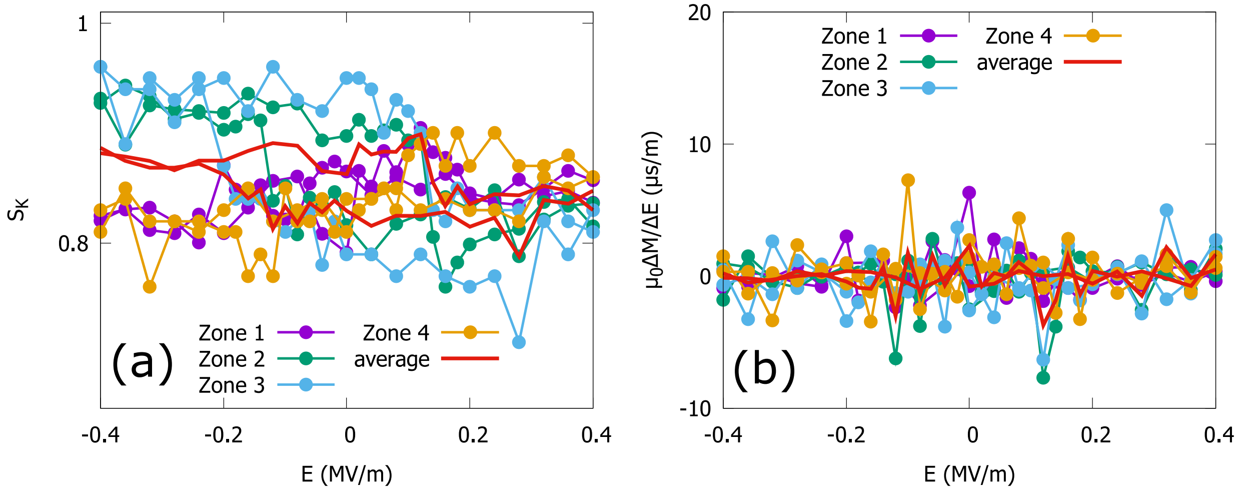

Figure S5: Magnetoelectric switch of $\mathbf{M}$ by the application of electric field in a polycrystalline FeGa film measured on four zones of the sample. (a) S_K *vs* E and (b) α_K *vs* E. The thick red lines correspond to the average over the four areas.

3.4 MOKE angular measurements

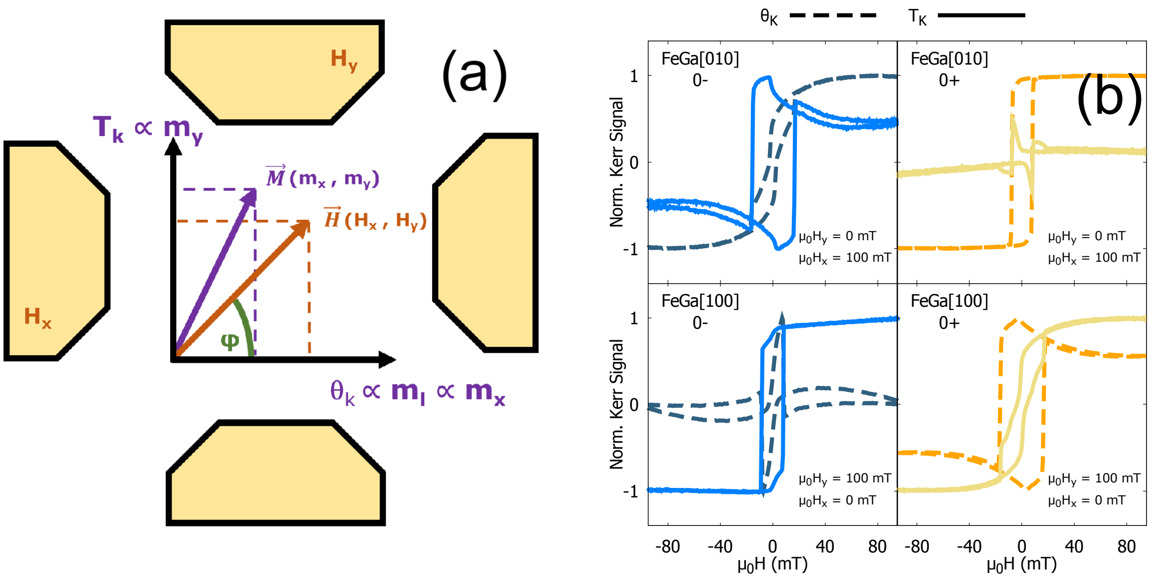

Figure S6: (a) Sketch of the MOKE setup. (b) θ_K and T_K for the FeGa(001) film obtained by means of MOKE magnetometry at the 0+ and 0- states for the H applied only along the x or y directions by means of a quadrupole magnet.

Obtaining the Kerr rotation loops *vs* φ introduces uncertainty due to the location of the laser spot during the rotation of the sample. The spot laser is not aligned with the rotation axis and a continuous readjustment of the beam is required to avoid wandering over the nonhomogenous FE substrate. We have opted to modify the angular position of the magnetic field by the use of a quadrupole magnet,[9] because the rotation of the magnet is not possible as indicated in the ROTMOKE method.[10] The sketch of this configuration is shown in Figure S6a.

The magnetic field $\mu_0 H_x$ is contained in the plane of incidence of the light and $\mu_0 H_y$ is along the transverse direction. Thus, θ_K and the transverse Kerr effect T_K signal are obtained by using a laser light with the p polarization. The magnetization component parallel to $\mathbf{H}$ is calculated as $m_x \cos\varphi + m_y \sin\varphi$, as can be deduced for the sketch in Figure S6a. To compare the θ_K and T_K signals, it is assumed that at the maximum applied field, θ_K and T_K correspond to the saturation values of the magnetization. The resulting coefficients are used

as calibration factor for all the cycles carried out as a function of H_x/H_y with the maximum applied field given by $\mu_0\sqrt{H_x^2 + H_y^2} = 100$ mT. Figure S6b shows normalized Kerr signals for $\mu_0 H_x = 100$ mT, $\mu_0 H_y = 0$ mT, and $\mu_0 H_x = 0$ mT, $\mu_0 H_y = 100$ mT, for the 0- and 0+ states of the FE structure. These measurements were used to obtain the calibration values. The influence of quadratic Kerr effects on the remanence value is eliminated by the average of the values at zero field for increasing and decreasing field sweeps.[8] Figure 4a in the main text shows the resulting $S_K(\varphi)$ curves with a clear uniaxial shape and the switching between easy and hard directions for the 0- and 0+ states.

4 180° switching of M

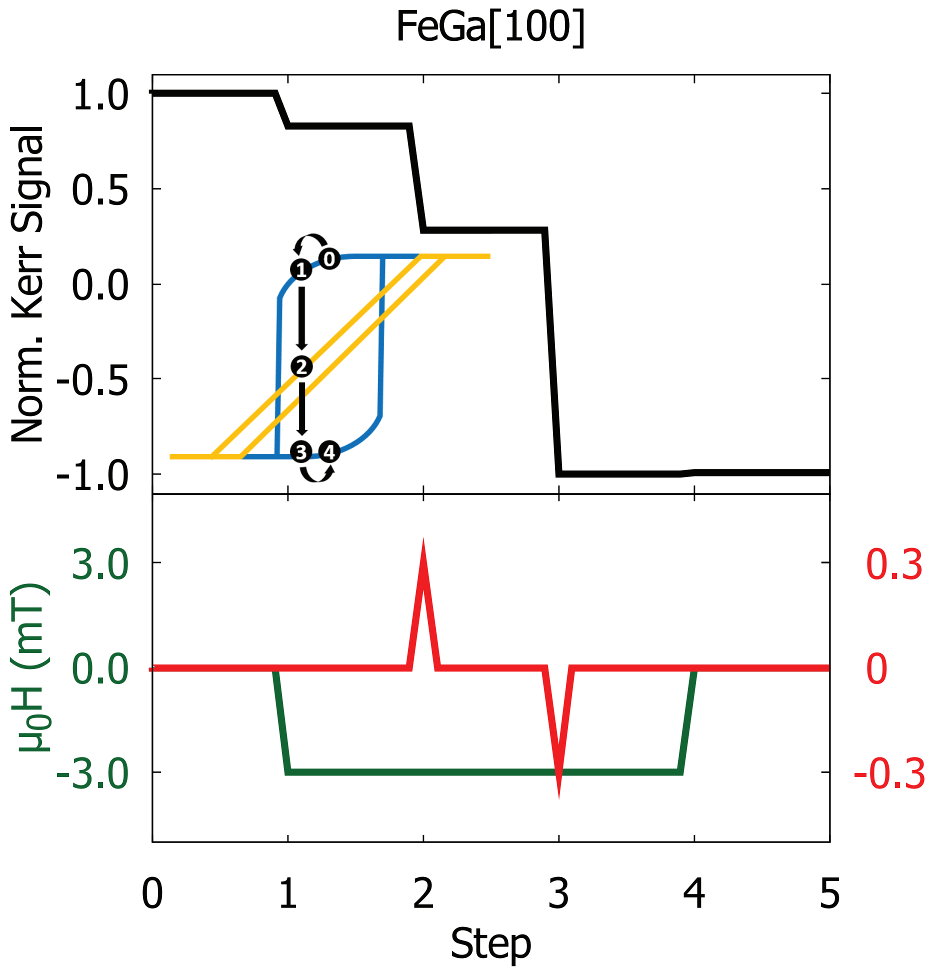

Figure S7: 180° switching

The 180° switching, from +M to -M, is obtained with the assistance of a magnetic field as low as 3 mT, by a procedure depicted in Figure S7. The black line in the upper panel of Figure S7 corresponds to the normalized longitudinal Kerr signal. The inset is a sketch of the hysteresis loops for the 0+ (yellow line) and 0- (blue line) states. The sequence to achieve 180° switching is:

Initial state (step 0): remanence at H = 0 and E = 0 after applying E = 0.3 M/V and H = 100 mT. The magnetic field has been applied along the easy direction induced by *E*.

Step 1: application of H = - 3 mT, in such a way that H is antiparallel to M.

Step 2: application of an electric pulse to switch the EA (blue MH loop) into HA (yellow MH loop) in Fig. S7. In this example with negative bias.

Step 3: application of an electric pulse to switch back the HA and EA. In this case with positive bias. Due to the presence of H, M and H become parallel to each other at this step.

Step 4: H is dropped out.

Step 5: M is at the remanence value.

5 Magnetic anisotropy energy

The expression for the magnetic anisotropy used in the main text,

$$e(\phi) = K_1 \sin^2\phi \cos^2\phi - B_1(\epsilon_{xx} - \epsilon_{yy}) \sin^2\phi + B_2\epsilon_{xy} \sin 2\phi \tag{S1}$$

can be obtained considering the cubic mangetocrystalline anisotropy:

$$e_{mc}(\phi) = K_1(\alpha_x^2\alpha_y^2 + \alpha_y^2\alpha_z^2 + \alpha_z^2\alpha_x^2) + K_2\alpha_x^2\alpha_y^2\alpha_z^2 \tag{S2}$$

with K_1 and K_2 first and second order magnetocrystalline constants and α_i the cosines of the magnetization. The magnetoelastic energy can be written as

$$\begin{aligned} e_{mel} &= B_1\left[\left(\alpha_z^2 - \frac{1}{3}\right)\left(\epsilon_{zz} - \frac{\epsilon_{xx}+\epsilon_{yy}}{2}\right) + \frac{1}{2}\left(\alpha_x^2 - \alpha_y^2\right)\left(\epsilon_{xx} - \epsilon_{yy}\right)\right] \\ &+ 2B_2\left(\alpha_x\alpha_y\epsilon_{xy} + \alpha_y\alpha_z\epsilon_{yz} + \alpha_z\alpha_x\epsilon_{zx}\right) \end{aligned} \tag{S3}$$

with B_1 and B_2 the irreducible cubic magnetoelastic coefficients.

We consider that the magnetization is confined in the plane so $\alpha_z = 0$, $\alpha_x = \cos\phi$, $\alpha_y = \sin\phi$. Thus the terms that maintain a dependence with ϕ correspond to those present in Equation S1.

References

(1) Nečas, D.; Klapetek, P. Gwyddion: an open-source software for SPM data analysis. *Open Physics* **01 Feb. 2012**, *10*, 181 – 188.

(2) Ciria, M.; Proietti, M. G.; Corredor, E. C.; Coffey, D.; Begué, A.; de la Fuente, C.; Arnaudas, J. I.; Ibarra, A. Crystal structure and local ordering in epitaxial $Fe_{100-x}Ga_x$/MgO(001) films. *J. Alloys Compd.* **2018**, *767*, 905–914.

(3) Thiele, C.; Dörr, K.; Bilani, O.; Rödel, J.; Schultz, L. Influence of strain on the magnetization and magnetoelectric effect in $La_{0.7}A_{0.3}MnO_3$/PMN-PT(001)(A=Sr,Ca). *Phys. Rev. B* **2007**, *75*, 054408.

(4) Cowburn, R. P.; Gray, S. J.; Ferré, J.; Bland, J. A. C.; Miltat, J. Magnetic switching and in-plane uniaxial anisotropy in ultrathin Ag/Fe/Ag(100) epitaxial films. *J. Appl. Phys.* **1995**, *78*, 7210.

(5) Mallik, S.; Chowdhury, N.; Bedanta, S. Interplay of uniaxial and cubic anisotropy in epitaxial Fe thin films on MgO (001) substrate. *AIP Adv.* **2014**, *4*, 097118.

(6) Postava, K.; Jaffres, H.; Schuhl, A.; Dau, F. N. V.; Goiran, M.; Fert, A. Linear and quadratic magneto-optical measurements of the spin reorientation in epitaxial Fe films on MgO. *J. Magn. Magn. Mater.* **1997**, *172*, 199 – 208.

(7) Clark, A. E.; Wun-Fogle, M.; Restorff, J.; Lograsso, T.; Cullen, J. Effect of quenching on the magnetostriction on $Fe_{1-x}Ga_x$ (0.13<x<0.21). *IEEE Trans. Magn.* **2001**, *37*, 2678–2680.

(8) Hamrle, J.; Blomeier, S.; Gaier, O.; Hillebrands, B.; Schneider, H.; Jakob, G.; Postava, K.; Felser, C. Huge quadratic magneto-optical Kerr effect and magnetization reversal in the Co_2FeSi Heusler compound. *J Phys D: Appl Phys* **2007**, *40*, 1563–1569.

(9) Li, J.; Jin, E.; Son, H.; Tan, A.; Cao, W. N.; Hwang, C.; Qiu, Z. Q. Design of a vector magnet for the measurements of anisotropic magnetoresistance and rotational magneto-optic Kerr effect. *Rev. Sci. Instrum.* **2012**, *83*, 033906.

(10) Mattheis, R.; Quednau, G. Determination of the anisotropy field strength in ultra-thin magnetic films using longitudinal MOKE and a rotating field: the ROTMOKE method. *J. Magn. Magn. Mater.* **1999**, *205*, 143 – 150.
